# 

**Published:** 1847-12

**Authors:** 


*To Readers, &c.*—The Editor and publishers of this Journal are often applied to for specimen numbers, that those who have some intention of patronising the work may examine it before subscribing. When this request is complied with, and the applicant concludes not to subscribe, the volume is broken, and it is all the same to the publishers as if the remaining numbers had been sent. On this account, it has become necessary to decline such applications. We would beg leave to suggest, that the best method of determining whether it is advisable to take the work, or not, is to subscribe for a year, and, at the end of that time, let it be discontinued if the subscriber be not satisfied.

We embrace this opportunity to say a word or two on other subjects relating to the Journal. And, first, we should be glad to receive a larger number of original communications. One great object in establishing the Journal was, to afford a convenient medium for interchanging views, and communicating facts appertaining to medicine and the medical profession.

We are well aware, that if this object were more fully carried out, it would add greatly to the interest and usefulness of the work. Practitioners, with whom we are casually thrown, frequently remark, that they have had it in contemplation to furnish articles, but have been deterred from so doing because it is not not done more generally by others. We would, therefore, urge all who are so disposed, not to wait for others to take precedence, but to make a beginning, and set an example, which, we do not doubt, will be followed, and prove advantageous to all parties. We say to our readers, one and all, write, that others may be encouraged to do so, and that you may have the benefit of their lucubrations ; *write*, that our science may receive advantage from the constant accession of new facts ; *write*, that medical periodical literature may be promoted ; and, last, not least, *write*, that you may derive personal satisfaction and profit therefrom, in the greater precision it will give to your ideas ; the stimulus to study, reflection, and observation it will afford ; and the greater interest it will cause you to feel in the daily exercise of your professional avocations. It is a secret worth knowing, that the most efficient of the external means to promote mental discipline, acquisition of knowledge, and scientific zeal, consists in making use of that humble, but potent instrument—the pen.

We trust our readers will excuse the bathos of passing abruptly to a topic of quite a different character, to wit : the payment of arrears. We are sorry to be obliged to allude again to this topic ; but there are still a few subscribers who have not paid for the first and second volumes of the Journal. We are compelled to know this, from the fact, that we are pecuniarily responsible for the amount which, through neglect, has not yet been received. Pray, bear this in mind, delinquents, and not ask us to pay your subscriptions, in addition to the editorial responsibilities which we have assumed for your benefit.

We must express our regrets that the work is not invariably punctual. If we could have our way, it would always be issued promptly on the first day of the month ; and it is but justice to ourselves to say, that, when it has not been so, it has been for reasons not within our control. We trust, that in future, there will be less occasion for apologies on that score.

We have reasons for the gratifying reflection, that there are not a few who take an interest in the prosperity of the Journal, and would be glad to promote its success. We would simply intimate, that efforts to extend its circulation will be the most effectual mode of securing its constant improvement, as well as giving a wider scope to whatever usefulness it may possess. It is evident that the circulation will be doubled at once, if each subscriber will secure another. We beg leave to commend this suggestion to those whose approval and good wishes we have been so fortunate to obtain in this enterprise.

